# Multiple Antimicrobial Resistance of *Escherichia coli* Isolated from Chickens in Iran

**DOI:** 10.1155/2014/491418

**Published:** 2014-12-07

**Authors:** Reza Talebiyan, Mehdi Kheradmand, Faham Khamesipour, Mohammad Rabiee-Faradonbeh

**Affiliations:** ^1^Department of Basis Sciences, Faculty of Veterinary Medicine, Islamic Azad University, Shahrekord Branch, P.O. Box 166, Shahrekord, Iran; ^2^Faculty of Veterinary Medicine, Islamic Azad University, Shahrekord Branch, Shahrekord, Iran; ^3^Young Researchers and Elite Club, Islamic Azad University, Shahrekord Branch, Shahrekord, Iran; ^4^Department of Microbiology, Islamic Azad University, Shahrekord Branch, Shahrekord, Iran

## Abstract

Antimicrobial agents are used extremely in order to reduce the great losses caused by *Escherichia coli* infections in poultry industry. In this study, 318 pathogenic *Escherichia coli* (APEC) strains isolated from commercial broiler flocks with coli-septicemia were examined for antimicrobials of both veterinary and human significance by disc diffusion method. Multiple resistances to antimicrobial agents were observed in all the isolates. Resistance to the antibiotics was as follows: Tylosin (88.68%), Erythromycin (71.70%), Oxytetracycline (43.40%), Sulfadimethoxine-Trimethoprim (39.62%), Enrofloxacin (37.74%), Florfenicol (35.85%), Chlortetracycline (33.96%), Doxycycline (16.98%), Difloxacin (32.08%), Danofloxacin (28.30%), Chloramphenicol (20.75%), Ciprofloxacin (7.55%), and Gentamicin (5.66%). This study showed resistance against the antimicrobial agents that are commonly applied in poultry, although resistance against the antibiotics that are only applied in humans or less frequently used in poultry was significantly low. This study emphasizes on the occurrence of multiple drug resistant *E. coli* among diseased broiler chickens in Iran. The data revealed the relative risks of using antimicrobials in poultry industry. It also concluded that use of antibiotics must be limited in poultry farms in order to reduce the antibiotic resistances.

## 1. Introduction 

Poultry production is one of the most important parts of farm industry in many countries including Iran. The high consumption of chicken meat requires great care to provide the safety of the industry against menacing factors [1]. Along with development of poultry farms and intensive culture, occurrence of the bacterial diseases and, consequently, overusing antibiotics have been increased in recent years.

Antimicrobials are valuable means to treat clinical disease and keep healthy and growth promotion. However, the treatment of all herds and flocks with antimicrobials for increasing the growth and preventing illness has become an endless debate [2]. Often whole flocks or herds of sick animals are treated at once, containing animals that are not sick. Moreover, antimicrobials are used in the absence of illness to prevent diseases when animals may be susceptible to infection [3]. This practice is very usual in Iran and other countries where outbreak is caused by enteric pathogens which are the sources of poultry farms diseases. Such a misuse and/or unsuitable usage affect a larger number of animals, since it usually interferes in treating the whole herd or flock again, which increases the possibility of selecting organisms resistant to the antibiotic. Furthermore, antimicrobial resistant pathogens also create a severe and costly animal health problem which may make the illness longer and reduce antimicrobial effectiveness leading to higher morbidity and mortality [4, 5].

In slaughterhouse, resistant strains from the gastrointestinal tract may infect chicken carcasses and, as a result, chicken meats are often related to multiresistant* E. coli*; also eggs become infected during laying [6]. Therefore, antimicrobial resistant fecal* E. coli* from poultry can infect humans directly and indirectly with food. Though seldom, these resistant bacteria may colonize in the human gastrointestinal tract and may also transfer resistance bacteria to human endogenous flora [7]. However, the mechanism of spreading the antimicrobial resistance transfer from animals' food to humans' remnants is contentious. Colonization of the gastrointestinal tract with resistant* E. coli* from broilers has been indicated in human volunteers [8]. There is historic evidence that animals are a source for* E. coli* found in humans. Resistance genes may be transferred vertically among bacteria of different genera and families or horizontally transferred between different bacterial species contained by the same genus or family and the possibility for transport of antimicrobial resistance genes among animals, humans, and the environment is a direct menace to public health [4].

The practice of using antimicrobials in feed may change the intestinal flora by posing a selective pressure in favor of resistant bacteria populations (such as resistant* E. coli*) that could find their path into the environment and food chain [9].

Data on the outbreak of antimicrobial resistant veterinary pathogens are required for knowledge based risk assessments concentrating on the relative risks concerning use of antimicrobial agents in animal treatment [10, 11].

This research aimed at investigating the antimicrobial resistance of* E. coli* living in broiler chickens breaded in Shahrekord Province, Iran.

## 2. Materials and Methods

### 2.1. Bacterial Isolates

Isolation and identification of* E. coli* were done by standard bacteriological methods. MacConkey and EMB agar were used for culturing of specimen and the colonies suspected of* E. coli* were identified by standard methods [12]. All strains of* E. coli* were isolated from 318 commercial broiler flocks, from April 2009 to March 2012 in Shahrekord Province, Iran. All of the samples were obtained from heart and liver of 7- to 14-day-old broiler chickens which suffered from septicemia in the past 24 hours.

### 2.2. Antimicrobial Susceptibility Determination

Antimicrobial susceptibility determination of isolated* E. coli* was completed by the standard disc diffusion procedure by taking into consideration the Clinical and Laboratory Standards Institute (CLSI) Performance Standards for Antimicrobial Disk Susceptibility Tests [13]. The* E. coli* strains were tested against the antibiotics of veterinary significance. The following antibiotic discs on Mueller Hinton agar were applied: Chloramphenicol (C/30 *μ*g), Chlortetracycline (CTe/30 *μ*g), Ciprofloxacin (CP/5 *μ*g), Danofloxacin (D/30 *μ*g), Difloxacin (DIF/25 *μ*g), Doxycycline (D/30 *μ*g), Enrofloxacin (NFX/5 *μ*g), Erythromycin (E/15 *μ*g), Florfenicol (FFc/30 *μ*g), Gentamicin (GM/10 *μ*g), Oxytetracycline (T/30 *μ*g), Sulfadimethoxine-Trimethoprim (SXT/25 *μ*g), and Tylosin (TYC/30 *μ*g).

## 3. Results

The highest rate of resistance was against Tylosin (88.68%), Erythromycin (71.70%), Oxytetracycline (43.40%), Sulfadimethoxine-Trimethoprim (39.62%), Enrofloxacin (37.74%), Florfenicol (35.85%), Chlortetracycline (33.96%), Difloxacin (32.08%), Danofloxacin (28.30%), Chloramphenicol (20.75%), and Doxycycline (16.98%). Low levels of resistance were against Ciprofloxacin (7.55%) and Gentamicin (5.66%). Susceptible (S), intermediate (I), and resistant (R) percentages of the isolates to the antimicrobial agents were showed in Table 1. Multiple resistances were observed in all of the isolates.

## 4. Discussion

*E. coli* is one of the most important factors of making economic losses resulting from diseases in commercial poultry farms and causing mortality as well as condemning the carcasses in slaughterhouses [14].

Antibiotics are the drugs used for preventing economic losses caused by* E. coli* and increasing the production efficiency [10]. But increasing consumption of these drugs leads to scattering them into manure and other poultry wastes and transferring them to humans by their remains in carcasses and can be the origin of bacterial resistances, mortality, and increase of the human hospitalization in hospitals [3, 15].

After a treatment by a selective pressure resulting from treatment by antibiotics, bacteria inside the body of diseased poultry tend to be changed into resistant strains. Through excretion and transferring of agricultural products by avian manure [16, 17] and direct transferring of resistant strains to humans using food chain, these strains lead to transferring the resistances, making the diseases caused by more expensive bacteria, increasing the time of treatment and mortality in human [1, 18, 19].

Increasing the aforementioned resistances motivated the governments to forbid the antibiotics consumption legally in order to keep the public health; therefore consumption of some antibiotics was forbidden including those which were regarded as growth stimuli in Europe and Furazolidone, Ciprofloxacin, and Chloramphenicol in Iran. Economic benefits resulting from poultry production have always been the motor of this industry. If no solutions for increasing the production efficiency and preventing economic losses resulting from coli bacillus are presented, the antibiotics consumption will be continued illegally [20].

It can be said that investigating the ways of transferring and prevalence of these resistances in poultry for better and optimal usage of these drugs is helpful. So, in this investigation, the resistances of the 7- to 14-year-old chickens of Shahrekord industrial poultry farm were studied from April 2009 to March 2012 and the following findings were obtained. Consistent with other researches, Gentamicin has the least resistance between the antibiotics consumed by poultry [21]. The reason is its low consumption in poultry due to its very low absorption by the digestive system of poultry and, consequently, its noneffectiveness [22].

In addition, in this examination, the resistance against Ciprofloxacin has been very low because of legal prohibition for consuming this antibiotic and its disuse in breeder farms. But previous surveys done at the final stage of production process of broiler chickens show that the resistance is very high which is because of illegal consumption of Ciprofloxacin [21].

The resistances against antibiotics of Chloramphenicol, Florfenicol, Enrofloxacin, Danofloxacin, Difloxacin, Oxytetracycline, Chlortetracycline, and Sulfadimethoxine-Trimethoprim have been obtained about 20 to 45 percent. Examining the past research, this difference is related to the time of sampling which emphasizes that the antibiotics consumption at the time of breeding creates a selective pressure for the bacteria in order for the resistant strains to be selected.

The high resistance of Erythromycin and Tylosin is also related to their impact on* Mycoplasma* which is highly used in breeders against* Mycoplasma synoviae* and* Mycoplasma gallisepticum*. Finally, from the findings of past studies, it can be concluded that incidence of resistances against antibiotics is different and increasing. For better usage and detecting the antibiotics, they should be tested by antimicrobial test. In is the only way upon which warranty the efficiency of the drug, consequently, the amount and frequency of antibiotic use is reduced, the economic benefits are accessible and spreading of antibiotic resistances are controllable.

## Figures and Tables

**Table 1:** Antimicrobial resistance and susceptibility of *E*. *coli* isolated from chickens (S) intermediate (I) resistant (R).

Antimicrobial agent	Diffusion zone breakpoint (mm)	Bacterial isolates (*n* = 318)					
		R		I		S	
		*n*	%	*n*	%	*n*	%
Aminoglycosides							
Gentamicin (GM/10 *μ*g)	≤12	18	(5.66)	39	(12.11)	261	(82.23)
Phenicols							
Chloramphenicol (C/30 *μ*g)	≤12	66	(20.75)	69	(21.65)	183	(57.60)
Florfenicol (FFc/30 *μ*g)	≤13	114	(35.85)	131	(41.31)	73	(22.84)
Quinolones							
Ciprofloxacin (CP/5 *μ*g)	≤15	24	(7.55)	45	(14.11)	249	(78.34)
Enrofloxacin (NFX/5 *μ*g)	≤17	120	(37.74)	71	(22.44)	130	(40.82)
Danofloxacin (D/30 *μ*g)	≤16	90	(28.30)	36	(11.37)	192	(60.33)
Difloxacin (DIF/25 *μ*g)	≤17	102	(32.08)	26	(8.33)	189	(59.59)
Tetracyclines							
Oxytetracycline (T/30 *μ*g)	≤14	138	(43.40)	123	(38.64)	57	(17.96)
Doxycycline (D/30 *μ*g)	≤13	54	(16.98)	135	(42.30)	129	(40.72)
Chlortetracycline (CTe/30 *μ*g)	≤14	108	(33.96)	92	(28.92)	118	(37.12)
Macrolides							
Erythromycin (E/15 *μ*g)	≤14	228	(71.70)	67	(21.04)	23	(7.26)
Tylosin (TYC/30 *μ*g)	≤14	282	(88.68)	36	(11.32)	0	(0.0)
Sulfadimethoxine-Trimethoprim (SXT/25 *μ*g)	≤10	126	(39.62)	75	(23.48)	117	(36.90)
